# A novel mutation in TNFRSF11A gene causes pediatric osteopetrosis: case report

**DOI:** 10.1186/s12893-021-01266-4

**Published:** 2021-05-28

**Authors:** You Xu, Xiaoyan Yu, Mengjie Huang

**Affiliations:** Department of Otolaryngology, Head & Neck Surgery, Chengdu Women’s and Children’s Central Hospital, School of Medicine, University of Electronic Science and Technology of China, Chengdu, 610091 China

**Keywords:** *TNFRSF11A* gene, Osteopetrosis, Autosomal recessive

## Abstract

**Background:**

Osteopetrosis is a rare inherited bone disorder affected individual by osteoclast disfunction and increasing bone density. Surgery was taken for histological examination of the specimen and evidence of malignancy was not found. Finally, X-ray and gene detection lead to the diagnosis.

**Case presentation:**

We report a 10-year-old girl with two years history of pus rhinorrhea, nasal obstruction and smelly nose. She was diagnosed and treated as sinusitis. But the symptoms were recurrent. Ten months ago, she was afflicted with persistent swelling and broken skin on the right cheek. All the laboratory findings showed normal. During surgery, we resected the right gingiva, the right nasal mucosa and the right facial tissue for biopsies. Histological examination showed proliferation of granulation tissue in chronic inflammatory mucosa. X-rays showed generalized sclerosis. Genetic analysis strongly supported a novel mutation of *TNFRSF11A* gene which caused osteoporosis. We found a novel mutation of the c.1196C > G (p.S399X) in exon 9 of *TNFRSF11A*. The *TNFRSF11A* gene encodes *RANK*, which is fundamental for osteoclast formation.

**Conclusion:**

Osteopetrosis is a rare genetic bone disease characterized by increased bone density because of bone resorption failure. Diagnosis is based on X-ray and gene analyze. Osteoclasts are bone-related cells derived from hematopoietic cell lines. Since osteoclasts arise from a hematopoietic progenitor cell of the monocytic lineage, the defect can be corrected by hematopoietic stem cell transplantation (HSCT). Better understanding of this pathological situation and pathogenesis is so important to plan appropriate immunotherapy to benefit.

## Background

Osteopetrosis is a rare inherited bone disorder characterized by osteoclast disfunction and increasing bone density [1]. It was first reported by a German radiologist named Albers-Schönberg in 1904.It was also called “Albers-Schönberg disease” or “marble disease”. This disease is caused by abnormal bone remodeling and resulted in osteoclast disorder. Osteoclasts are bone-related cells derived from hematopoietic cell lines. Increase in bone density leads to limitation of bone marrow cavity, resulted in hematopoietic failure and anemia or pancytopenia, followed by hepatosplenomegaly and frontal bone eminence. Expanding bone will have a negative impact on the patient's movement, and lead to blindness, deafness, even facial paralysis and dysphagia [2].

According to the basis of mode of inheritance, osteopetrosis is generally classified into autosomal recessive osteopetrosis (ARO), autosomal dominant osteopetrosis (ADO) and X-linked [3–6]. ARO is now documented to affect 1 in 250 000 live births, ADO is with an incidence of approximately 1 in 20,000 births [7, 8]. Osteopetrosis exhibits high phenotypic and genotypic diversity, patients with different mutation can be symptomatic or asymptomatic, with symptoms various from mild to severe. Due to clinical manifestation, the disease can be divided into three types: mild, intermediate and severe types. The severe type has increased bone density and frequent fractures. To the contrary, as for the mild group, some of which have normal life expectancy [9]^.^

Infantile malignant osteopetrosis is the most several type of autosomal recessive osteopetrosis. It is usually detected during infancy or early childhood due to whole body bone sclerosis, short stature, macrocephaly, tooth deformities, hepatosplenomegaly, severe anemia, and persistent fracture [10]. Although approximately 30% of the etiology of human osteopetrosis remain genetically unrecognized, but so far, at least 20 genes mutation including *TCIRG1*, *CLCN7*, *SNX10*, *OSTM1*, *PLEKHM1*, *CAII*, *FERMT3*,*RANKL*, *RANK( TNFRSF11)*, *SLC29A3*, *TRAF6*,*LRRK1*,*MITF*, *NEMO*, *RELA*,*CSF1R*,*C16ORF57*,*CTSK*,*Kindlin-3*,*CalDAG-GEFI*, have been reported to associated with human osteopetrosis [3–6]. *TCIRG1* is the most common one. Here we present a case of a pediatric female, who carried a homozygous gene mutation in *TNFRSF11A*.

## Case presentation

**Fig. 1:**
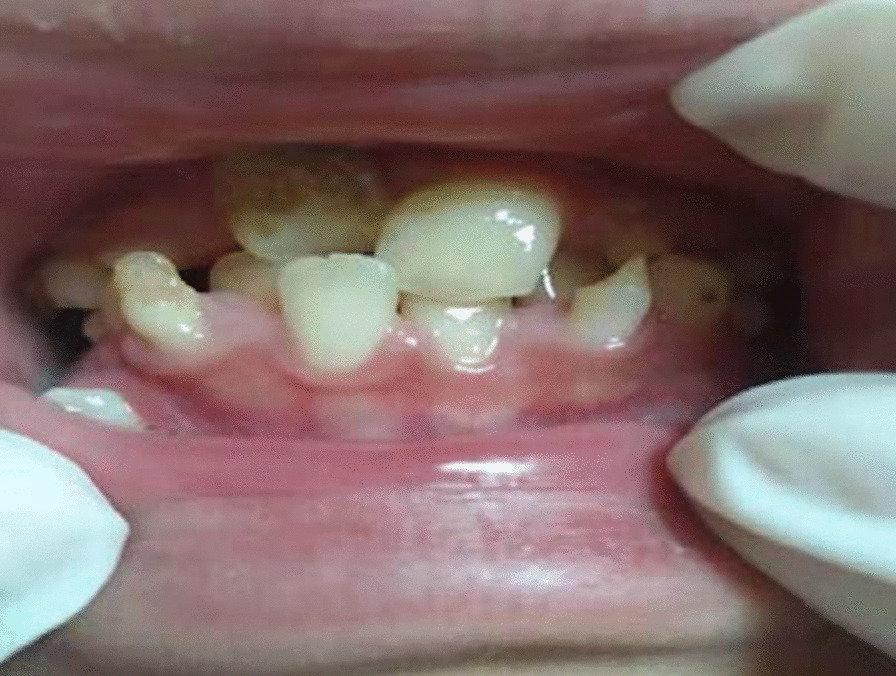
Intraoral front view- mixed dentition and caries

**Fig. 2:**
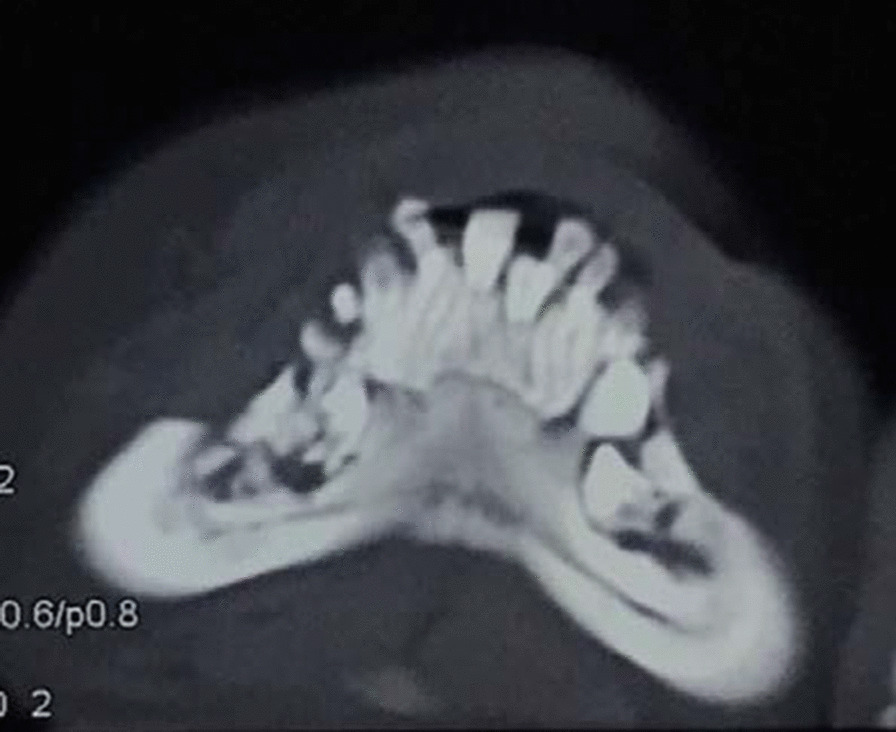
Destruction of maxillary alveolar bone

A 10-year-old girl was referred by her doctor with the chief complains of pus rhinorrhea, nasal obstruction and smelly nose for two years. Ten months ago, she was afflicted with persistent swelling and broken skin on the right cheek. Physical examination showed that smelly purulent secretion of abscess along with soft tissues below right medial canthus. Poor nasal ventilation, secretion occupied middle meatus and total meatus. Several teeth did not erupt and were still sub-gingival. So it was difficult to distinguish primary and permanent teeth (Fig. 1). #84,85 root malformations and alveolar bone osteonecrosis were seen (Fig. 2). Oral hygiene was poor; rampant caries and gingivitis were present.

**Fig. 3:**
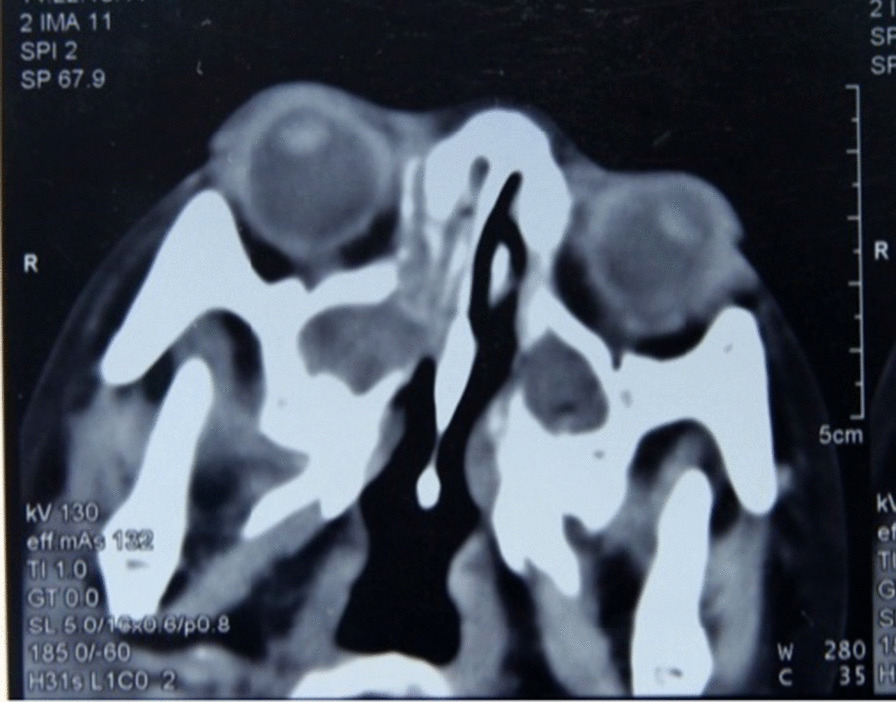
Paranasal sinusitis

The girl stayed as an inpatient for 24 days. In hospital, antibiotic of amoxicillin and clavulanate potassium (800 mg per 8 h) was using for 7 days and the skin of her cheek began to heal. For evaluation the cause of the disease, laboratory tests; computer tomography (CT) of sinusitis and surgery was taken. All the laboratory findings showed normal. CT of and paranasal sinus shows bone destruction of right maxillary sinus wall and right nasal bone, and maxillary sinus and anterior part of nasal cavity are filled with more soft tissue density (Fig. 3). On day 8, surgery was carried out under general anesthesia. During surgery, we resected the right gingiva, the right nasal mucosa and the right facial tissue for biopsies. Histological examination showed proliferation of granulation tissue in chronic inflammatory mucosa. Evidence of malignancy was not observed.

**Fig. 4:**
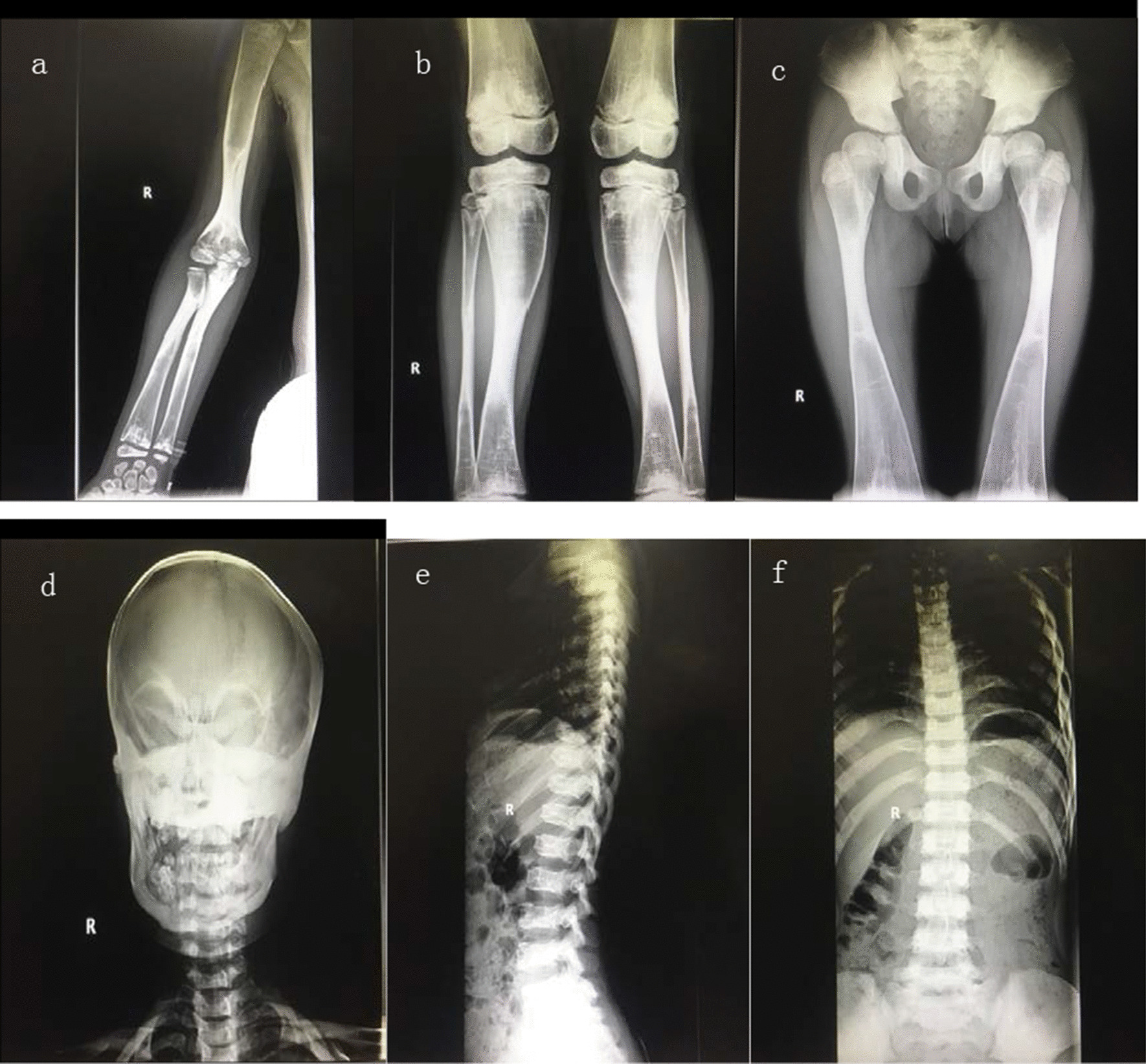
**a**–**c **The structure of limb bones is deformed. Extreme metaphyseal flaring, reduced bone vascularity and dense bone structure is found. **d **Skull radiograph showing typical wolf carnival appearance. **e**, **f** Front-Lateral Chest radiograph showing diffuse sclerotic vertebra and costochondral junction widening

All the examination results could not lead to diagnosis. After the hospital consultation, we suspected the diagnosis of osteopetrosis. X-rays of hands, legs, chest and skull was taken place on day 15. Radiographs showed multiple bony abnormalities involving the skull, vertebrae and limb. X-rays showed generalized sclerosis, for example: thickening and increasing Cranial plate, narrowing diploic vein, Sella turcica, increased vertebral bone density in cliff, smaller pituitary fossa, and costochondral junction widening. A typical form of “sandwich” vertebra was noted. Longitudinal striation of the long bones, extreme metaphyseal flaring, reduced bone vascularity and dense bone structure was simply found (Fig. 4).

After informed consent, Peripheral blood samples (3 ml) from the proband, sister and both parents were collected into graded negative pressure vacuum ethylene diamine tetraacetic acid (EDTA)anticoagulant tubes. Genomic deoxyribonucleic acid (DNA) from all available family members were obtained for Sanger sequencing. The whole‐exome sequencing and Sanger sequencing were performed by Beijing Mygenostics Co, Ltd were taken place. Targeted gene panel sequencing was performed to check for the presence of pathogenic variants of multiple associated genes responsible for osteopetrosis.

**Fig. 5:**
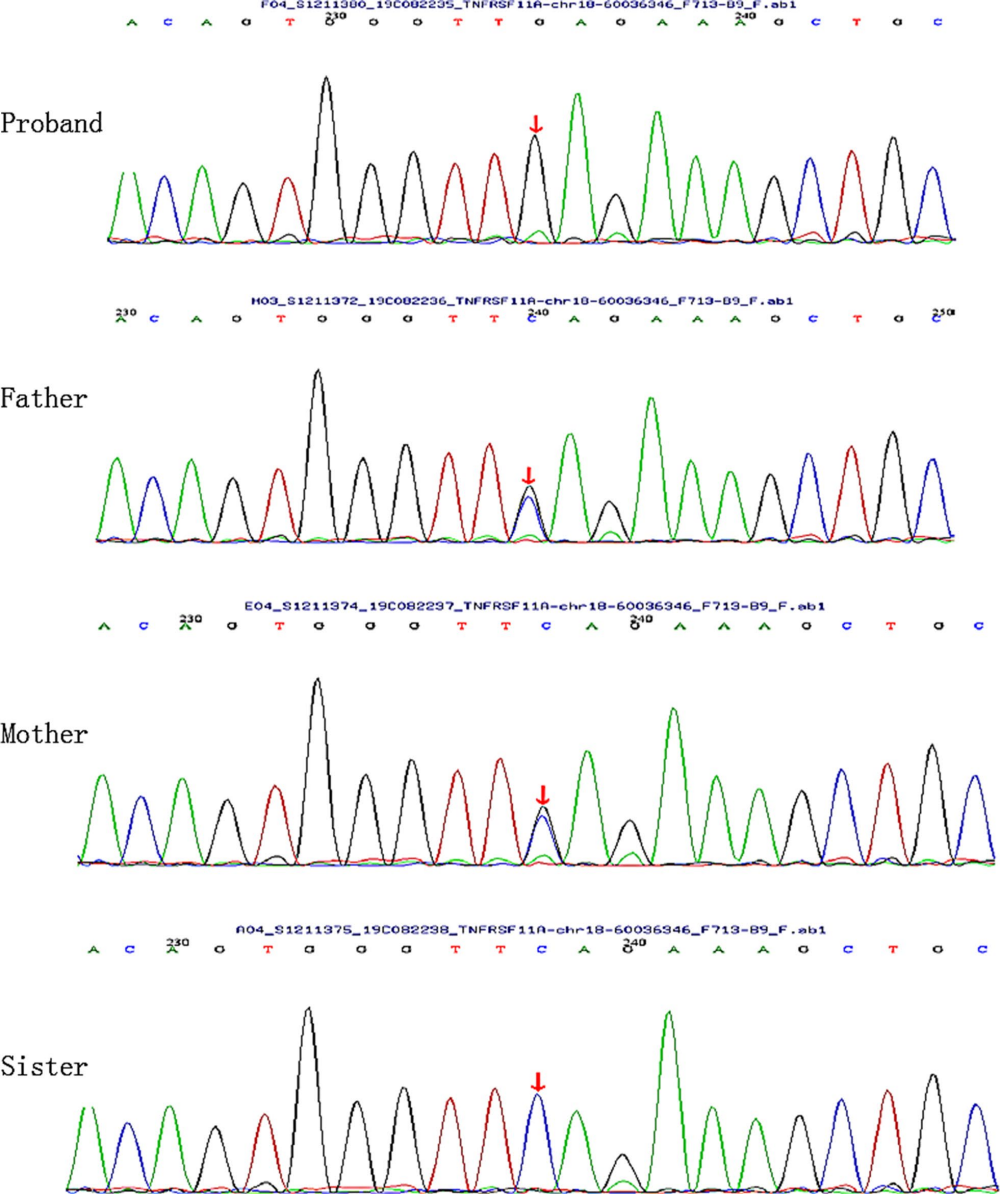
A homozygous nonsense mutation of the c.1196C > G (p.S399X) of the TNFRSF11A gene was identified in the proband and marked with arrow. The same heterozygous variant was found in both the patient’s father and mother. The sister is normal

The results of whole‐exome sequencing revealed homozygous nonsense mutation c.1196C > G (p.S399X) in exon 9 of the *TNFRSF11A* gene in the proband. It was a novel mutation which was not previously reported in a patient with osteopetrosis. Sanger sequencing was applied to confirm the presence of this identified variant, and the same heterozygous variant was found in both the patient’s father and mother (Fig. 5). However, the parents denied having any complaints including osteomyelitis, history of fracture, visual impairment and hearing loss.

## Discussion and conclusion

In this case, the child went to the otolaryngology department with the onset of nasal symptoms as the main-complain. After systemic X-ray examination with characteristic imaging changes and genetic test, the patient was diagnosed of osteopetrosis. This is the first case of pediatric osteopetrosis in China with a mutation in *TNFRSF11A* gene. The molecular analysis of the patient led to the identification of a novel mutations in the *TNFRSF11A* gene: a nonsense mutations c.1196C > G (p.S399X) in exon 9.Mutations in the human *TNFRSF11A* gene which leading to osteopetrosis was first reported by Sobacchi [11]. Monogenic mutations of *TNFRSF11A* has previously been fully documented as the cause of human autosomal recessive osteopetrosis [12–14]. It caused amino acid substitutions at moderately conserved position and supporting the diagnosis of autosomal recessive osteopetrosis type 7 [15].

*TNFRSF11A*(*RANK)* gene belongs to the tumor necrosis factor (TNF) receptor superfamily and located on chromosome 18 (18q21.33), which encoding a receptor with 616 amino acids [16]. The *RANK* gene codes for the functional receptor for *RANKL*. The mutated residues seem to be highly conserved in evolution, so it could be considered that amino acid substitutions at these positions might change the folding of the outer domain thus the interaction with *RANKL*. *RANKL*, *RANK* and *TNFRSF11* affect the differentiation of osteoclasts, resulting in a decrease in the number of osteoclasts, while the remaining genes all cause osteoclast dysfunction [17].

*RANK*-dependent ARO is confirmed to benefit from HSCT, although there is no long-term follow-up data. In the post-HSCT period, patients seem to be particularly prone to hypercalcemia, especially when HSCT surgery is older. These data increase the clinical and molecular heterogeneity of human ARO, further confirming the important role of accurate molecular diagnosis in therapy.

Until now, *TNFRSF11A* mutations have been observed in patients with the Paget’s disease of bone (PDB), primary ovarian insufficiency, recurrent fevers, familial expansile osteolysis, expansile skeletal hyperphosphatasia and autosomal recessive osteopetrosis [2, 16]. The *TNFRSF11A* gene encodes *RANK*, which is fundamental for osteoclast formation. This has been demonstrated before, which impacted osteoclasts maturation and have a severe defect in bone resorption and remodeling.

At present, there is no acceptable best therapy for osteopetrosis, as a result, symptomatic and supportive treatment was given to most patients [18]. HSCT is the only treatment that can offer cure to these patients according to reports, but not all the subtypes can benefit from HSCT (e.g. *OSTM1* and some with *CLCN7*) [13]. Osteopetrosis can be diagnosed by typical x-ray manifestation. Typical bony abnormalities were illustrated, including generalized sclerosis, longitudinal striation of the long bones, extreme metaphyseal flaring or transverse lucent bands, “sandwich” vertebra, flask deformities, “bone-within-bone” appearance. Bone marrow biopsy showed shrinkage of bone marrow cavity, thickening of bone trabecula, reduction of bone marrow hematopoietic cells, and bone marrow fibrosis [9, 19].

The clinical manifestations included rhinitis, alveolar bone osteonecrosis, osteomyelitis of alveolar bone, bone destruction of right maxillary sinus, failure of tooth eruption, roots malformation, caries, optic nerve atrophy, compressive neuropathies, cranial nerve deficit [2]^.^ The child exhibited most of the phenotypic who was diagnosed after two years. She is the first case of osteopetrosis in the family, her parents and old sister are with normal bone density. Her parents are close relatives.

Clinic examination of ear nose and throat (ENT) doctors combining the whole-body radiographic results can lead to early diagnosis of osteopetrosis. Vigorous physical activities should be avoided to prevent severe complications. Thus, routine ENT and dental examination should be applied to prevent sinusitis and osteomyelitis as well. The role of *TNFRSF11A* mutations in osteopetrosis has been revealed not long ago. Genetic analysis strongly supported the pathogenic role for this mutation. Better understanding of this pathological situation and pathogenesis is so important to plan appropriate immunotherapy to benefit. In summary, we report a novel mutation of *TNFRSF11A* gene in a Chinese girl with osteopetrosis. Analysis and reporting patients with mutation in this gene can be very helpful to obtain a better picture of the disease phenotype in *TNFRSF11A*-related osteopetrosis.

## Data Availability

Available at the request of the readers.

## Abbreviations

HSCT: Hematopoietic stem cell transplantation
ARO: Autosomal recessive osteopetrosis
ADO: Autosomal dominant osteopetrosis
EDTA: Ethylene Diamine Tetraacetic Acid
DNA: Deoxyribonucleic acid
CT: Computed tomography
TNF: Tumor necrosis factor
PDB: Paget’s disease of bone
ENT: Ear nose and throat
